# Interpersonal variability of the human gut virome confounds disease signal detection in IBD

**DOI:** 10.1038/s42003-023-04592-w

**Published:** 2023-02-25

**Authors:** Stephen R. Stockdale, Andrey N. Shkoporov, Ekaterina V. Khokhlova, Karen M. Daly, Siobhan A. McDonnell, Orla O’ Regan, James A. Nolan, Thomas D. S. Sutton, Adam G. Clooney, Feargal J. Ryan, Donal Sheehan, Aonghus Lavelle, Lorraine A. Draper, Fergus Shanahan, R. Paul Ross, Colin Hill

**Affiliations:** 1 APC Microbiome Ireland, University College Cork, Co, Cork, Ireland; 2 Department of Medicine, University College Cork, Co, Cork, Ireland; 3 School of Microbiology, University College Cork, Co, Cork, Ireland

**Keywords:** Bacteriophages, Gastrointestinal diseases

## Abstract

Viruses are increasingly recognised as important components of the human microbiome, fulfilling numerous ecological roles including bacterial predation, immune stimulation, genetic diversification, horizontal gene transfer, microbial interactions, and augmentation of metabolic functions. However, our current view of the human gut virome is tainted by previous sequencing requirements that necessitated the amplification of starting nucleic acids. In this study, we performed an original longitudinal analysis of 40 healthy control, 19 Crohn’s disease, and 20 ulcerative colitis viromes over three time points without an amplification bias, which revealed and highlighted the interpersonal individuality of the human gut virome. In contrast to a 16 S rRNA gene analysis of matched samples, we show that α- and β-diversity metrics of unamplified viromes are not as efficient at discerning controls from patients with inflammatory bowel disease. Additionally, we explored the intrinsic properties of unamplified gut viromes and show there is considerable interpersonal variability in viral taxa, infrequent longitudinal persistence of intrapersonal viruses, and vast fluctuations in the abundance of temporal viruses. Together, these properties of unamplified faecal viromes confound the ability to discern disease associations but significantly advance toward an unbiased and accurate representation of the human gut virome.

## Abstract (web summary)

A longitudinal analysis of unamplified fecal virome of healthy controls and IBD patients reveals interpersonal variability is a factor that limits our ability to identify associations between the virome composition and IBD.

## Introduction

The microbiome of patients with inflammatory bowel disease (IBD) has been the subject of frequent investigations as it is deemed a central component of disease pathogenesis^1–3^. Assessing the intra- and interpersonal variations in bacterial composition through 16S rRNA gene amplicon sequencing has become a keystone of microbiome studies and it has been shown that bacterial diversity is often indicative of disease activity and severity, particularly in IBD^4^. In recent years, the viral constituents of the human microbiome have gained recognition for their potentially important role in the maintenance of health and wellness. Previous studies have observed alterations in the diversity, composition, and/or functionality of the gut virome associated with a range of conditions, including type 1 and type 2 diabetes, irritable bowel syndrome, and IBD^5–10^.

As highlighted by Gregory et al.^11^ during their compilation of a human gut virome database, 96% of the studies they analysed employed multiple displacement amplification (MDA). This was included in the vast majority of studies to provide sufficient DNA for metagenomic sequencing. However, when applied to viromes, MDA selectively amplifies small circular and single-stranded DNA viruses, is associated with chimeric sequence formation, and under amplifies GC-rich genomes with non-uniform amplification of linear genomes^12–14^. Recent improvements in sequencing technology mean difficult-to-achieve starting quantities of nucleic acids are no longer an issue, eliminating the MDA step previously required. Therefore, it is an opportune and appropriate time to re-analyse the IBD virome, particularly considering the conflicting conclusions previously reported.

Many of the contradictions reported between IBD virome studies can be attributed to inconsistent methodological and computational approaches. For example, Norman et al.^15^ concluded that the richness of tailed phages was increased amongst patients with Crohn’s disease (CD) and ulcerative colitis (UC) relative to controls. This was later refuted by Clooney et al.^16^ who included “viral dark matter” sequences with no database representatives. Zuo et al.^17^ believed the mucosal virome of patients with UC contained a high viral load of low diversity phages including giant viruses. The presence of giant viruses was later argued by Sutton et al.^18^ to result from flawed taxonomic assignment approaches. Finally, Manrique et al.^19^ proposed there was a globally distributed healthy gut phageome of core and common viruses that was diminished in patients with IBD. However, this concept was thrown into doubt by the immense diversity and interpersonal variability of human-associated viruses observed in subsequent meta-analyses of Earth’s virome^20–23^.

The study reported here performed an original longitudinal analysis of the human gut, analysing both the virome of healthy controls and patients with IBD, without an MDA step, in combination with matched 16S rRNA amplicon sequencing. Our analysis of unamplified faecal viromes confirms that modest but significant alterations are observed in the intra- and interpersonal diversity metrics of IBD gut viromes compared to controls. However, 16S rRNA amplicon sequencing of corresponding samples showed much more clearly the distinct intra- and inter-sample diversity features frequently observed in IBD. During our investigation of unamplified viromes as potential biomarkers of IBD, we concluded that the lack of a disease signal could be attributed to (i) the infrequent detection of interpersonal communal viruses, (ii) the prevalence of transiently detected intrapersonal viruses and (iii) the temporal fluctuations of intrapersonal persistent viruses, as compared to bacterial taxa. This analysis highlights the interpersonal variability of the human gut virome and the need for the viral research community to establish stringent and consistent criteria when discerning associations between viruses and biomarkers of health and disease.

## Results

### An overview of unamplified faecal viromes

In this study, we analysed unamplified faecal viromes to perform one of the most unbiased assessments of the viral components of the human microbiome to date. A total of 40 healthy controls and 39 patients with IBD donated two (*n* = 4) or three (*n* = 75) faecal samples at approx. 100-day intervals (T2: mean 93.2, SD 28.8; T3: mean 192.3, SD 71.7; see Supplementary Data). Virome samples were sequenced across four runs, with the randomisation of control, CD, and UC samples per run resulting in no significant difference in the final number of reads obtained per condition (Supplementary Fig. 1a–c). A significant decrease in the number of viral-recruited reads (to the viral contig database created in this study) per sample was observed for patients with IBD, with a concomitant increase in bacterial and human-recruited reads (Supplementary Fig. 1d–h). Extensive metadata encompassing 88 variables were collected from patients with IBD, with Supplementary Fig. 2 providing a simplified overview of the cohorts’ lifestyle choices and medical history (for further information, see Supplementary Data).

**Fig. 1:**
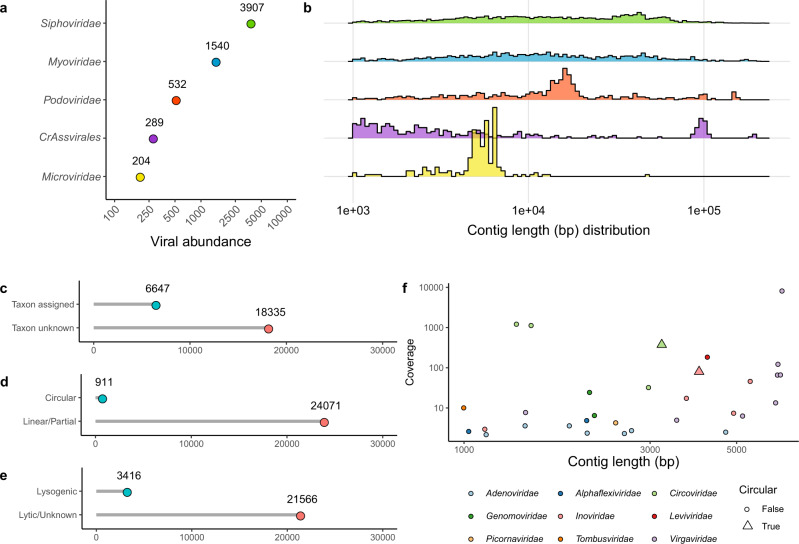
Overview of unamplified faecal viromes detected in control and IBD subjects. **a** The frequencies of the most abundant viruses assigned familial taxonomic ranks, showing **b** their contig length distributions. The number of viruses identified in this study that (**c**) could be assigned a taxonomic rank, **d** characterised as circular (True = circular; False = non-circular partial sequence/linear), or **e** encoding genes for phage lysogenic replication. **f** The contig length relative to sequence coverage for infrequently detected eukaryotic faecal viruses that could be assigned a taxonomic rank.

Similar to previous gut virome studies^24^, strict filtering criteria were applied to viral enriched metagenomes to remove potential contaminants during analyses (Supplementary Fig. 3). Out of 65,852 viral contigs, 18,149 represented previously reported viral species (with ≥95% nucleotide identity and ≥85% coverage cut-off)^25^ from recent comprehensive surveys of the human gut virome^21,26^. The rest of the viral contigs were unique to the present study. The majority of the taxonomically identifiable human faecal virus are tailed phages of the class *Caudoviricetes* (94.3%), historically exemplified as families *Siphoviridae*, *Myoviridae*, and *Podoviridae* (Fig. 1a). Unsurprisingly, human-associated *Crassvirales* phages with characteristic ~100 kb circular genomes were prominent within faecal viromes (Fig. 1b). As expected, the unamplified gut virome is dominated by “unclassified viruses”. Specifically, only 26.6% and 13.7% of this study’s gut virome database could be assigned taxonomic or life cycle information, respectively (Fig. 1c, e). Even without MDA, this study identified 911 circular viral genomes (Fig. 1d) including 112 circular *Microviridae* genomes. Human-host and plant-infecting viruses constitute only a small fraction of the total faecal viruses detected (Fig. 1f). For instance, eleven genomes of plant RNA viruses (*Alphaflexiviridae*, *Tombusviridae*, *Virgaviridae*), apparently of dietary origin, were detected. In addition, a single enterovirus genome (*Picornaviridae*) and several human adenovirus genome fragments were detected. However, in contrast to previous studies employing MDA^27,28^, relatively few eukaryote-infecting viruses with small circular genomes (CRESS DNA viruses^29^,) were identified (e.g., *Anelloviridae* and *Circoviridae*).

### Differences in unamplified virome and 16S diversity metrics in IBD

**Fig. 2:**
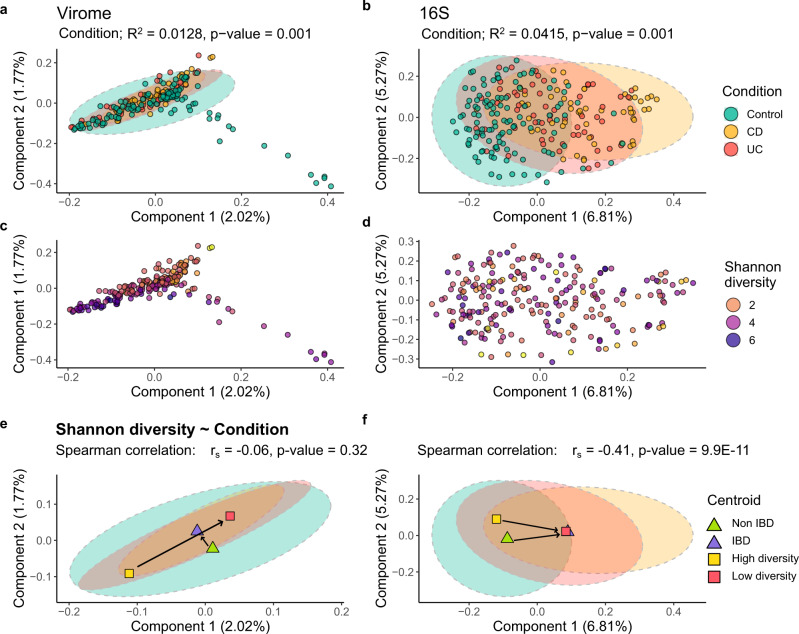
Diversity analyses of unamplified faecal viromes. PCoA β-diversity ordinations, using Canberra distances, highlighting compositional variation within **a** unamplified viromes and **b** 16S rRNA gene compositions, grouped by condition. Again, β-diversity ordinations of **c** unamplified virome and **d** 16S data, but each individual sample is coloured by its Shannon index α-diversity value. PCoA centroids for control and IBD sample ordinations, and the top 10 most and least α-diverse samples, for the **e** unamplified virome and **f** 16S ordinations. Spearman’s correlation (r_s_) and *p*-values are shown above (**e**, **f**), calculating the association of sample α -diversity to condition. Spearman’s correlation coefficients (+/−): 0.0–0.19 = very weak; 0.40–0.59 = moderate. Statistics are based on *n* = 118 control samples, *n* = 56 CD samples and *n* = 59 UC samples.

While distinctly visible in the 16S β-diversity analysis, no clear ordination separation of controls versus patients with CD or UC is discernible using unamplified virome data (Fig. 2a, b). Indeed, a statistically smaller variance (R^2^) is attributable to the condition variable for the virome data, compared to 16 S (PERMANOVA *p* ≤ 0.001, 1.28% and 4.15%, respectively). Like previously reported^30,31^, α-diversity analyses of 16 S data showed a clearly significant reduction in the diversity, evenness, and richness metrics associated with CD and UC samples compared to control samples (Supplementary Fig. 4b). However, differences in the α-diversity of unamplified viromes are less pronounced with only the richness of control samples versus CD samples showing a clear significant difference (*p* = 0.008; Supplementary Fig. 4a).

Superimposing the α-diversity of individual samples onto the β-diversity PCoA ordinations tentatively indicated that 16 S differences in α-diversity occur along the same β-diversity PCoA axis as disease separation (Fig. 2c, d). To highlight this result more clearly and determine if the same was true for the virome data, the β-diversity PCoA centroids for control and IBD patients were calculated alongside the centroids for the ten highest and lowest α-diversity samples. For the 16S data, there is a clear convergence of centroids from the highest α-diversity/non-IBD samples to the lowest α-diversity/IBD samples (Fig. 2f). Whereas for unamplified viromes, α-diversity and IBD status are juxtaposed (Fig. 2e). This observation was supported using multiple β-diversity distance metrics (Supplementary Fig. 5).

Finally, while the Shannon index α-diversity of 16 S samples shows a moderate but significant correlation with condition, no significant correlation is observed for unamplified viromes (Fig. 2e, f). Furthermore, only “very weak” and “weak” insignificant correlations are observed between the α-diversities of unamplified virome and 16 S samples, even when stratified by condition (Supplementary Fig. 6).

### The individuality of faecal viromes is more extreme in IBD

**Fig. 3:**
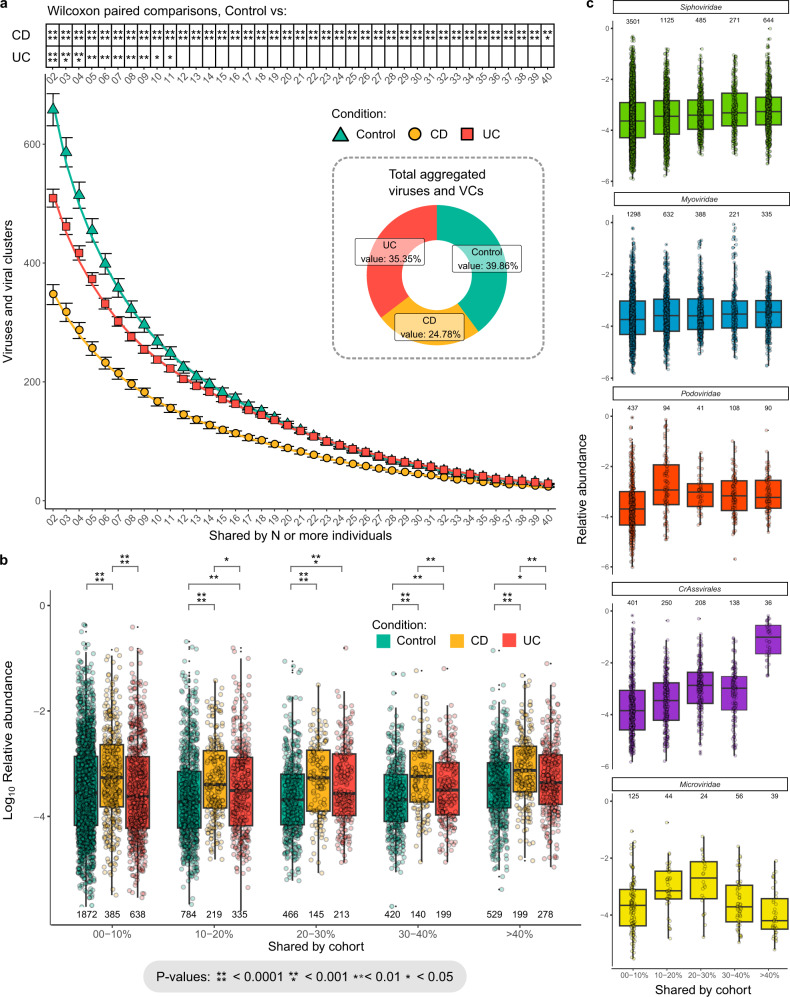
Communal and compositional analyses of unamplified faecal viromes. **a** The number of communal viruses and viral clusters (VCs) shared by an increasing number of control, CD, and UC individuals. Bootstrapped subsampling of a single time point from 10 random individuals, for each condition, was performed 20 times. The relative abundance of faecal viruses, demonstrating **b** lysogenic phages shared between increasing percentages of control, CD, and UC individuals (Student’s *t* test was used to calculate statistical differences between cohorts), and **c** the prevalence of dominant viral taxa shared amongst increasing percentages of the total cohort individuals. The values at the top of each boxplot indicates the number of viruses or VCs averaged. Boxplots represent the standard Tukey representation, with boxes representing the 25th, 50th (median) and 75th interquartile range (IQR) percentiles, and the whiskers encompassing values within 1.5 times the IQR.

Despite the limited differences observed in the α-diversities of controls and patients with IBD, there were large disproportional changes in the number of viral taxa shared between cohort members. To avoid introducing a bias by sampling cohorts of differing sizes, first, the number of viruses or viral clusters (VCs) shared across individuals (henceforth termed “communal” viruses) was calculated. Subsequently, 10 individuals from each condition were randomly selected 20 times and the frequency at which the previously identified communal viruses occurred was calculated. Immediately it is evident that there is a significantly greater chance for controls to possess viruses or VCs that are shared by two or more individuals and therefore their viromes are not as individual-specific (Fig. 3a). The observation that different cohorts harbour communal viruses to an altered extent became insignificant between control and UC individuals when viruses or VCs present in more than eleven of the 79 cohort members were considered. However, there remained a significant difference in the detection of communal viruses between controls and CD patients across >50% of the total study’s population.

Next, we focused our analysis on the fraction of human faecal viruses that were capable of a temperate lifestyle due to the presence of gene(s) associated with a lysogenic lifecycle. Previous reports found an increase in the presence of temperate phages in the faeces of patients with IBD, possibly the result of an inflamed gut environment inducing lysogenic phages^16^. We similarly observed an increase in the average relative abundance of phages harbouring lysogenic genes in patients with IBD relative to controls, particularly when viruses were stratified by their degree of sharing across the total cohort (Fig. 3b). However, as before, the number of viruses carrying lysogenic genes and VCs present in the unamplified faecal viromes of patients with CD and shared by >40% of the total cohort is fewer than amongst controls (199 versus 529, respectively).

When unamplified faecal viromes are analysed with respect to assignable taxonomic information, there are both predictable and novel features associated with viruses and VCs shared across the study’s total cohort. As expected, *crAssvirales* are frequently observed in human faeces and constitute a large proportion of the average relative abundance of viromes (Fig. 3c)^32–36^. In contrast, the average relative abundance of *Siphoviridae* shared by the study population is more evenly distributed across all strata investigated. Unexpectedly, there are discrete relative abundance peaks associated with communal viruses shared across the total cohort for *Myoviridae* (30–40%), *Podoviridae* (10–20%) and *Microviridae* (10–20% and 20–30%). Therefore, while not as universally present in viromes as the crAss-like phages, there are potentially discrete distributions of putative *Myoviridae*, *Podoviridae* and *Microviridae* viruses or VCs within human microbiome populations.

Finally, we analysed the cumulative versus the average relative abundance of communal viruses shared by an increasing number of this study’s cohort. As the majority of unamplified faecal viruses or VCs are uniquely or rarely shared by cohort individuals (0–10%) they have the greatest accumulative relative abundance, but viruses or VCs frequently encountered in viromes (>40%) have the greatest average relative abundance (Supplementary Fig. 7a). Of note, communal viruses or VCs in controls vs patients with CD (0–10%) reached statistical significance (Supplementary Fig. 7b). For the remaining unshared, less frequently encountered unamplified gut viruses with taxonomic information identified, most are only sporadically detected in cohort members (Supplementary Fig. 7c). An exception is dietary plant viruses of the *Virgaviridae* family that are frequently detected (30–40%).

### Temporal variability is a feature of faecal viromes

**Fig. 4:**
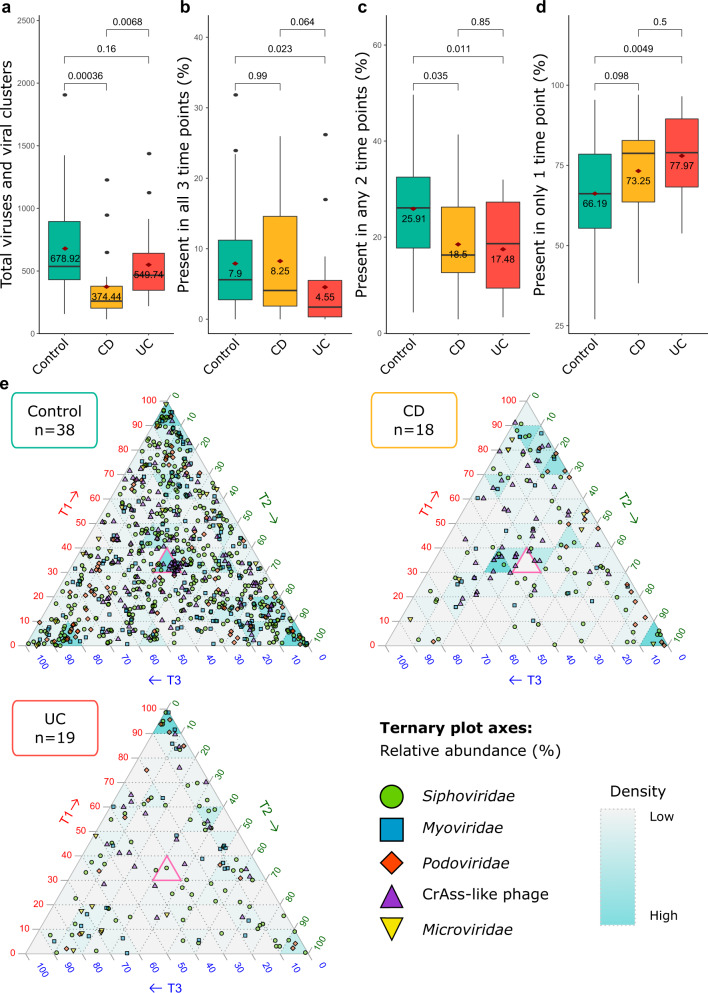
Temporal stability of unamplified faecal viromes. **a** The total number of viruses and viral clusters (VCs) detected across control, CD, and UC faecal viromes where three time points could be analysed (*n* = 225 samples). The percentage of viruses and VCs detected in **b** all three time points (*n* = 225 samples), **c** two time points (*n* = 233 samples), and **d** unique to only one time point (*n* = 233 samples). Wilcoxon test p-values for specific group comparisons are shown. Boxplots represent the standard Tukey representation, with boxes representing the 25th, 50th (median) and 75th interquartile range (IQR) percentiles, and the whiskers encompassing values within 1.5 times the IQR. Red diamonds with values underneath display the mean. **e** Ternary plots showing the relative abundance, as a percentage, of viruses and VCs with taxonomic assignments across the three time points. Viruses and VCs with a relative abundance of 33% (pink triangle) were equally present across all three time points. The shape aesthetics indicates putative viral familial assignments, while the background shading of the ternary plot triangles represents viral positional density.

Variability in the human gut virome has clear implications when discerning potential associations of viruses or VCs that co-occur with microbial or physiological biomarkers. Using our unamplified faecal virome data, we set about characterising the persistence and fluctuations of viruses or VCs longitudinally. When the average number of viruses or VCs detected per faecal virome by condition is analysed without using rarefied data, the richness of control viromes is greater than that of patients with IBD (Fig. 4a). Statistically, fewer viruses or VCs persist across all three time points of UC viromes compared to the viromes of controls and patients with CD (Fig. 4b). However, both CD and UC viromes had statistically fewer viruses present in two of the three time points when compared to controls (Fig. 4c). Finally, patients with CD and UC had the greatest percentage of unique viruses or VCs present in only one of their three time points (Fig. 4d).

While 16S and virome sequencing data can provide different views of the microbiome, we can look at how bacterial and viral taxa are shared across healthy individuals or patients with IBD to determine if there are notable differences in these cohorts. The number of 16S communal taxa shared by cohort members gradually decreases for controls and patients with CD and UC (Supplementary Fig. 8a). However, there is a noticeable drop-off in shared 16S communal taxa by CD and subsequently UC microbiomes, as the criterion for sharing viruses, that is presence of viruses across individuals is increased (by approx. 20 and 25 individuals, respectively). For viromes, there is a rapid decrease in the sharing of communal viruses or VCs by controls and patients with IBD (Supplementary Fig. 8b). In all instances, Control viromes share the most communal viral taxa, followed by UC and finally CD viromes. Despite the clear starting difference in the number of 16S and viral taxa, the interpersonal variability of the virome in this study results in little sharing of taxa across cohort members.

Fluctuations in the relative abundance of viruses or VCs present at all three time points were performed (Fig. 4e). There were 38 Control, 18 CD and 19 UC viromes where all three time points were available. Viruses located in the central pink triangle of each ternary plot represent viruses or VCs with an equal relative abundance, expressed as a percentage, across the three time points (i.e., 33% on each axis). It is striking that (a) the greater number of viruses present in the Control ternary plot, and (b) the seemingly random fluctuation of viruses or VCs across the three time points. The latter is influenced by both the greater number of control samples available for analysis and the increased richness associated with controls, while the former is true for both viruses with and without taxonomic information (Fig. 4e and Supplementary Fig. 9, respectively). To discern if there is a statistical difference between intra- and interpersonal unamplified gut viromes, the dissimilarity of viromes was calculated using the Bray-Curtis index. Interestingly, while no intra-personal variability is discernible using the matched 16S sample data, there is a statistically significant difference in both the intra- and interpersonal virome (Supplementary Fig. 10).

## Discussion

Recent gut microbiome studies are demonstrating bacteria and their phages co-exist in stable equilibrium, lasting for many months and even years^24,37–39^. Such equilibrium is achieved through convergence of different ecological and co-evolutionary mechanisms operating at the level of individual phage-host pairs (evolutionary arms race, fluctuating selection) and at the level of complex polymicrobial community as a whole (kill-the-winner dynamics, host jumps). This complex web of interactions may lead to a situation where both the phages and their hosts mutually benefit from each other’s presence in the system, termed “antagonistic co-evolutionary mutualism” in the recent review^39^. Phages may improve the fitness and resilience of their host bacteria populations as predators, driving diversifying selection^40,41^, or as lysogens altering phenotypic properties^42^. Understanding these complex interactions, particularly to the point of correctly discerning and/or modulating health or disease-associated properties, remains a significant challenge for those studying the human microbiome and its viral constituents.

In this study, we compared unamplified faecal viromes alongside 16S rRNA gene analysis of matched samples, contrasting healthy controls and patients with CD and UC. Even when we generated viral clusters from assembled viral strains to resemble the taxonomic rank of 16S OTUs, unamplified viromes and 16S rRNA gene amplicons are different datatypes that represent different aspects of the microbiome. Therefore, direct comparisons need to be performed and interpreted carefully. Indeed, all virome and 16S rRNA gene comparisons that are described in this study were performed with the same diversity metrics and are presented, where possible, side-by-side. We believe the analysis presented, in contrast to prior landmark studies that employed MDA^15,16,19,24,27,43–48^, is a progressive step towards an unbiased understanding of human microbiomes and the most accurate assessment of healthy and IBD viromes to date. The inter-individual variability of virome is a known fact demonstrated in previous studies by our group^16,24^ becomes even more prominent when shotgun sequencing methods are used which avoid biases of MDA. A principal reason for that is that the virome is fundamentally analysed at strain level. High strain-level diversity of bacteriophages, rapid evolution of their genomes, lack of evolutionary-conserved genes, high levels of genome mosaicism, lack of correspondence between phage taxonomy and taxonomy of their bacterial hosts create staggering levels of phage diversity in the human gut, within and between individual human subjects, even twins^44^. With advances in database collation of viral data and taxonomic classification (higher-order phage taxa introduced in recent revision of ICTV taxonomy) and genome-based grouping of viruses (vConTACT2 and similar approaches) we hope to gather better insight into their biological function/correlation with disease phenotypes.

The α-diversity of unamplified gut viromes, compared to 16 S rRNA genes, is poor at differentiating healthy controls from patients with IBD. Marginally significant results (assuming p < 0.05) are seen between specific groups with respect to their intra-sample diversity, evenness, and richness metrics. However, clear α-diversity differences are seen in the 16 S analysis of controls versus patients with IBD, which subsequently translates into interpersonal microbiome variations. The inconsistency between virome and 16 S rRNA gene α-diversity reflects the intrinsic characteristics of the human gut microbiome, whereby healthy viromes can consist of a few dominant viruses or a multitude^37^.

Manrique *et al*. (2016) previously proposed a “healthy gut phageome” was composed of globally distributed core and common viruses^19^. With hindsight, their conclusions of a worldwide core virome is at odds with the vast diversity and interpersonal variability of human faecal viromes. However, given the advances in sequencing technologies and viral databases, we chose to further investigate the concept that fewer viruses are shared between patients with IBD. Initially, due to the high intra- and inter-individuality of gut viromes, we determined which viruses within our database were potentially gut-specific and communal, i.e., shared by two or more viromes. Our analysis of unamplified faecal viromes demonstrated that control viromes were significantly more likely to be composed of communal viruses compared to IBD viromes. Furthermore, communal viruses with an identifiable gene responsible for temperate replication were shared to a greater degree by patients with IBD, particularly CD. Therefore, within discrete geographic locations and disease states, there are likely gut-specific communal viruses shared by populations that differ in their genotypic and phenotypic characteristics, such as replication strategy.

Building upon a previous hypothesis by Shkoporov et al. (2020) that gut microbiomes have a core persistent personal virome (PPV) while the majority are transiently detected^24^, we compared the longitudinal carriage of viruses by controls and patients with IBD. Indeed, the majority of gut viruses are present only in a single time point analysed, with IBD viromes containing more viruses unique to each time point. The core PPV of controls and patients with IBD, spanning approx. 200 days, constituted less than 10% of their overall detected viromes. Therefore, studies investigating gut viromes over longer timeframes, or even over consecutive days, would help develop our understanding of the stability of viruses within PPVs.

Finally, given that our analysis of gut viromes was performed without MDA, we wanted to conduct an assessment of the fluctuations of viruses comprising PPVs without an amplification bias. For both controls and patients with IBD, the relative abundance of longitudinally persistent viruses fluctuates seemingly randomly between time points. Additionally, the intra-personal dissimilarity of gut viromes was more pronounced for patients with IBD. However, recent virome sequencing studies have included a spike-in control for conducting absolute quantifications of viruses^38^. Therefore, future gut virome analyses utilising optimised and standardised procedures have the potential to resolve ambiguities of the gut virome that will help generate a more holistic model of the human gut microbiome in health and disease. This will be further enhanced by the curation and taxonomic classification of viruses in our publicly available databases.

Previous gut virome studies employed MDA to obtain sufficient DNA for sequencing. Amusingly, however, the use of MDA in gut virome studies could be considered a double-edged sword. While MDA undoubtedly biases the true composition of viromes, it also reduces the interpersonal variability of gut viromes. Our analysis of unamplified gut viromes shows intrapersonal α-diversity metrics are limited in their discrimination of healthy control and IBD viromes. Furthermore, while endeavouring to detect viral biomarkers associated with IBD, we identified three major factors hampering disease signal detection. Firstly, there is a high compositional variability of viromes between individuals. Secondly, many gut viruses are only transient. And finally, the abundance of longitudinally persistent viruses fluctuates dramatically. Considering these complex properties of viromes, correctly identifying associations between viral taxa and disease biomarkers will be significantly more challenging than for 16 S rRNA gene analyses. However, a better understanding of all constituents of human microbiomes is nonetheless required before targeted interventions could become a possible treatment for complex gastrointestinal diseases.

## Methods

### Faecal sample collection and nucleic acid sequencing

Patients with IBD were recruited to donate faecal samples for microbiome analysis through a speciality IBD clinic, run by experienced physicians. Control subjects were enroled in study protocol APC055, which was approved by the Clinical Research Ethics Committee of the Cork Teaching Hospitals. All methods were carried out in accordance with relevant guidelines and regulations. Informed consent was obtained from all adult donors with a written questionnaire completed to partake in the study. Relevant clinical data and characteristics were recorded for controls and recruited patients, including basic lifestyle information (Supplementary Table 1), disease activity for patients with IBD (Supplementary Table 2), and generic and IBD-specific medications (Supplementary Table 3). For a more complete overview of this study’s metadata resource, see Supplementary Data.

Faecal samples were collected from volunteers without additives or preservatives, transported to the research facility at ambient temperature, and were stored at −80^o^C until processed. Virus-like particle (VLP) extraction was performed from, 0.5 g faeces resuspended in 10 mL of SM buffer, mixed by vigorous vortexing for 5 min. Samples were then cooled on ice for 5 min prior to centrifugation at 5000 rpm in a swing bucket rotor for 10 min at + 4 °C. Supernatants were decanted into new tubes, and centrifugation was repeated. The resulting supernatants were then filtered twice through a 0.45-μm pore PES syringe-mounted membrane filters. NaCl and PEG-8000 powders were then added to the filtrates to give a final concentration of 0.5 M and 10% w/v, respectively. Following complete dissolving, samples were incubated overnight (16 h) at + 4 °C.

On the following day, the samples were centrifuged at 5000 rpm for 20 min at + 4 °C to collect the precipitate. The supernatant was discarded, and tubes were inverted on paper towels for 5 min to remove any remaining liquid. Pellets were then resuspended in 400 μl of SM buffer and gentle shaken with an equal volume of chloroform. Emulsions were then centrifuged at 2500 g for 5 min using a desktop centrifuge. The aqueous phase (~ 360 μl) was pipetted into a clean Eppendorf tube and mixed with 40 μl of a solution of 10 mM CaCl_2_ and 50 mM MgCl_2_. After addition of 8 U of TURBO DNase (Ambion/ThermoFisher Scientific) and 20 U of RNase I (ThermoFisher Scientific) free DNA/RNA digestion was carried out at 37 °C for 1 h before inactivating enzymes at 70 °C for 10 min. Proteinase K (40 μg) and 20 μl of 10% SDS were then added, and incubated for 20 min at 56 °C. Finally, viral particles were lysed using 100 μl of Phage Lysis Buffer (4.5 M guanidinium isothiocyanate, 44 mM sodium citrate pH 7.0, 0.88% sarkosyl, 0.72% 2-mercaptoethanol) with incubation at 65 °C for 10 min. Lysates were then extracted twice by gentle shaking with equal volume of Phenol/Chloroform/Isoamyl Alcohol 25:24:1 (Fisher Scientific) followed by centrifugation at 8000 g for 5 min at room temperature. The resulting aqueous phase was subjected to final round of purification using DNeasy Blood & Tissue Kit (Qiagen) according to manufacturer’s instruction with a final elution volume of 50 μl. The concentration of viral nucleic acids were assessed using the Qubit dsDNA HS kit (ThermoFisher Scientific). Extracted VLPs yielded an average DNA concentration of 3.99 ng/µl (see Supplementary data for individual sample concentrations). Subsequently reverse transcription of potential RNA viral genomes was performed, and 100 nanograms of each purified DNA sample was sheared with M220 Focused-Ultrasonicator (Covaris) applying the 350 bp DNA fragment length settings (peak power 50 W, duty factor 20%, 200 cycles per burst, total duration of 65 s). Sequencing libraries were subsequently created using the Accel-NGS 1 S Plus DNA library kit (Swift Biosciences). Ready-to-load libraries were sequenced using 2 × 150 nt paired-end sequencing runs on an Illumina HiSeq 2500 platform at GATC Biotech AG, Germany. Similar methodology was previously used by our group for virome studies^49^.

### Computational analysis

The treatment of raw VLP and 16S rRNA gene sequencing data followed established pipelines^24,38,49^. Briefly, for 16S rRNA amplicon data processing, paired-end reads were merged and filtered using *a* < 0.5 expected error rate per nucleotide and total length. Reads were dereplicated and singletons removed, following the trimming of the forward and reverse primers (“-stripleft 17” and “-stripright 21”, respectively). OTUs were clustered at 97% identity and reference-based chimera removal was performed using UCHIME. OTUs were assigned taxonomic information by aligning reads to the RDP Gold database using the RDP Classifier (v2.12)^50^.

The VLP sequencing data was manipulated as follows. Read quality, adaptor removal, and quality trimming (SLIDINGWINDOW:4:20 MINLEN:60 HEADCROP:10) was performed using FastQC (v0.11.5), cutadapt (v1.9.1), and TrimmomaticPE (v0.36), respectively^51–53^. Levels of bacterial and human contamination in the VLP sequencing data were estimated using Bowtie2 alignments against a *cpn60* gene database and through Kraken alignments against the reference human genome GRCh38, respectively^54,55^. Contigs were assembled using SPAdes (v3.11) in metagenomic mode^56,57^, with short (<1 kb) and redundant (90% identity over 90% length) contigs discarded. Open reading frames were predicted using Prodigal (v2.6.3) in metagenomic mode with Shine-Dalgarno training disabled. The detection of putative viruses within the VLP sequencing data was performed as described previously^58^ in a manner that avoids inclusion of potential bacterial contaminants. Briefly, contig-encoded proteins were queried against the Prokaryotic Viral Orthologous Groups database (pVOGs) using HMMER version 3.1.b252. The following cut-offs were employed to detect sequences rich in viral proteins: contigs <5 kb needed ≥3 pVOG hits; ≥5 and <10 kb, 4 pVOGs; ≥10 and <20 kb, 5 pVOGs; ≥20 and <40 kb, 6 pVOGs; ≥40 and <60 kb, 7 pVOGs; and ≥60 kb, 8 pVOGs. Sequences identified through the different approaches were pooled together and made nonredundant, keeping the larger of two sequences when the BLAST identity and coverage between sequences exceeded 90%.

Viruses were deemed truly present within a sample, and not a spurious detection, when ten or more reads with a SAMTools (v0.1.19)/BEDTools (v2.26.0) calculated breadth of coverage for Bowtie2 mapped reads spanned 50% of contigs <5 kb, 30% of contigs ≥5 kb and <20 kb, or 10% of contigs ≥20 kb^59,60^.

The final read counts of the virome and 16 S rRNA gene analyses, with accompanying metadata, were imported into R Studio (v3.6.1) for analysis^61^. Dataframes and matrices were manipulated, as necessary, using the reshape2 package^62^. Read counts were converted into relative abundances using the funrar package^63^. Images were generated using ggplot2 with the ggpubr extension^64,65^. Colour palettes were sourced from the RColorBrewer, pals, and viridis packages^66–68^. The taxonomic information for putative viruses, encompassing both historic and incumbent terms, were generated using Demovir (https://github.com/feargalr/Demovir). Intra- and inter-personal diversity metrics were calculated using vegan and phyloseq^69,70^. The α-diversity metrics presented are the Shannon index for diversity, Pielou’s J for evenness, and rarified richness for species richness. The β-diversity distances were calculated using Canberra distances, unless otherwise stated, with two-dimensional ordination of samples employing PcoA. The PCA analysis was generated using the base R stats package^71^. Ternary plots were created using the Ternary package^72^.

### Statistics and Reproducibility

Analysis is based on 233 samples (*n* = 118 control samples, *n* = 56 CD samples and *n* = 59 UC samples) donated by volunteers at up to 3 time points. Patients with IBD and Control subjects were enroled in study protocol APC055, which was approved by the Clinical Research Ethics Committee of the Cork Teaching Hospitals. All methods were carried out in accordance with relevant guidelines and regulations. Informed consent was obtained from all adult donors in the study.

Graphical representation of analyses includes boxplots, which represent the standard Tukey representation, with boxes representing the 25th, 50th (median) and 75th interquartile range (IQR) percentiles, and the whiskers encompassing values within 1.5 times the IQR. Bar plots depict mean values with error bars representing the standard deviation. Student T-test or the Wilcoxon test were employed to determine statistical difference between two specific groups. Statistical significance was assumed as a *p*-value ≤ 0.05, with false discovery rate adjustments employing Bonferroni correction. Centroids were calculated as the mean location of data points with regard the relevant axes. Permutational multivariate analysis of variance (PERMANOVA) statistical tests were calculated using the adonis function of vegan. Associations between diversity values were calculated using Spearman’s correlation through the base R stats package. Individual images were organised into their final multi-panel display using Inkscape (v 1.1.2).

### Reporting Summary

Further information on research design is available in the Nature Portfolio Reporting Summary linked to this article.

## Supplementary information

Supplementary Figures and Tables

Description of Additional Supplementary Files

Supplementary data

NR reporting summary

## Data Availability

The data and scripts required to generate the images and interpret the results of this study are provided as Supplementary Data. The virome and 16S rRNA amplicon sequencing data analysed in this study is available through NCBI BioProject code: PRJNA828396.
