# Supplementary material for: Interpersonal variability of the human gut virome confounds disease signal detection in IBD
Source: Commun Biol. 2023 Feb 25;6:221. doi: 10.1038/s42003-023-04592-w (PMC9968284; doi:10.1038/s42003-023-04592-w)
Supplement: Supplementary file 1 — Supplementary Figures and Tables [file 42003_2023_4592_MOESM1_ESM.pdf]

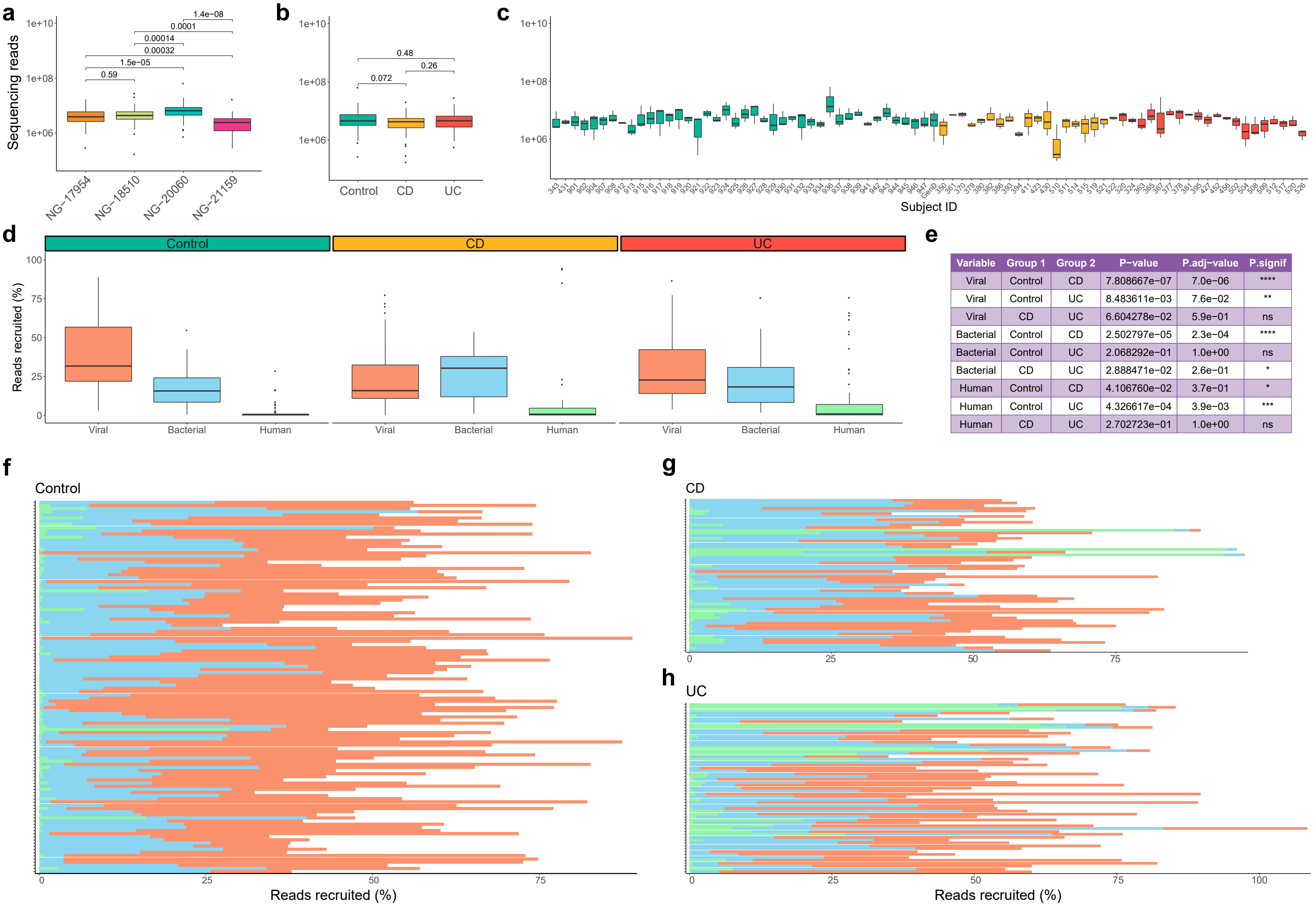

Variable	Group 1	Group 2	P−value	P.adj−value	P.signif
Viral	Control	CD	7.808667e−07	7.0e−06	****
Viral	Control	UC	8.483611e−03	7.6e−02	**
Viral	CD	UC	6.604278e−02	5.9e−01	ns
Bacterial	Control	CD	2.502797e−05	2.3e−04	****
Bacterial	Control	UC	2.068292e−01	1.0e+00	ns
Bacterial	CD	UC	2.888471e−02	2.6e−01	*
Human	Control	CD	4.106760e−02	3.7e−01	*
Human	Control	UC	4.326617e−04	3.9e−03	***
Human	CD	UC	2.702723e−01	1.0e+00	ns

Supplementary Figure 1. Overview of the virome sequencing reads generated in this study. (a) Sequencing reads per faecal virome segregated by sequencing run. Wilcoxon p-values are shown between specific comparisons. **(b)** Virome sequencing reads by host condition, and **(c)** per individual subject. **(d)** The percentage of faecal sample sequencing reads recuited to the predicted virome, and the estimated percentages of bacterial and human contamination. **(e)** Wilcoxon paired statistical comparisons of percentage reads recruited, grouped by Condition. An overview of the percentage of reads recruited to viral, bacterial, and human sources, separated by **(f)** Control, **(g)** CD, and **(h)** UC Condition. Statistics are based on n=118 control samples, n=56 CD samples and n=59 UC samples. Boxplots represent the standard Tukey representation, with boxes representing the 25th, 50th (median) and 75th interquartile range (IQR) percentiles, and the whiskers encompassing values within 1.5 times the IQR.

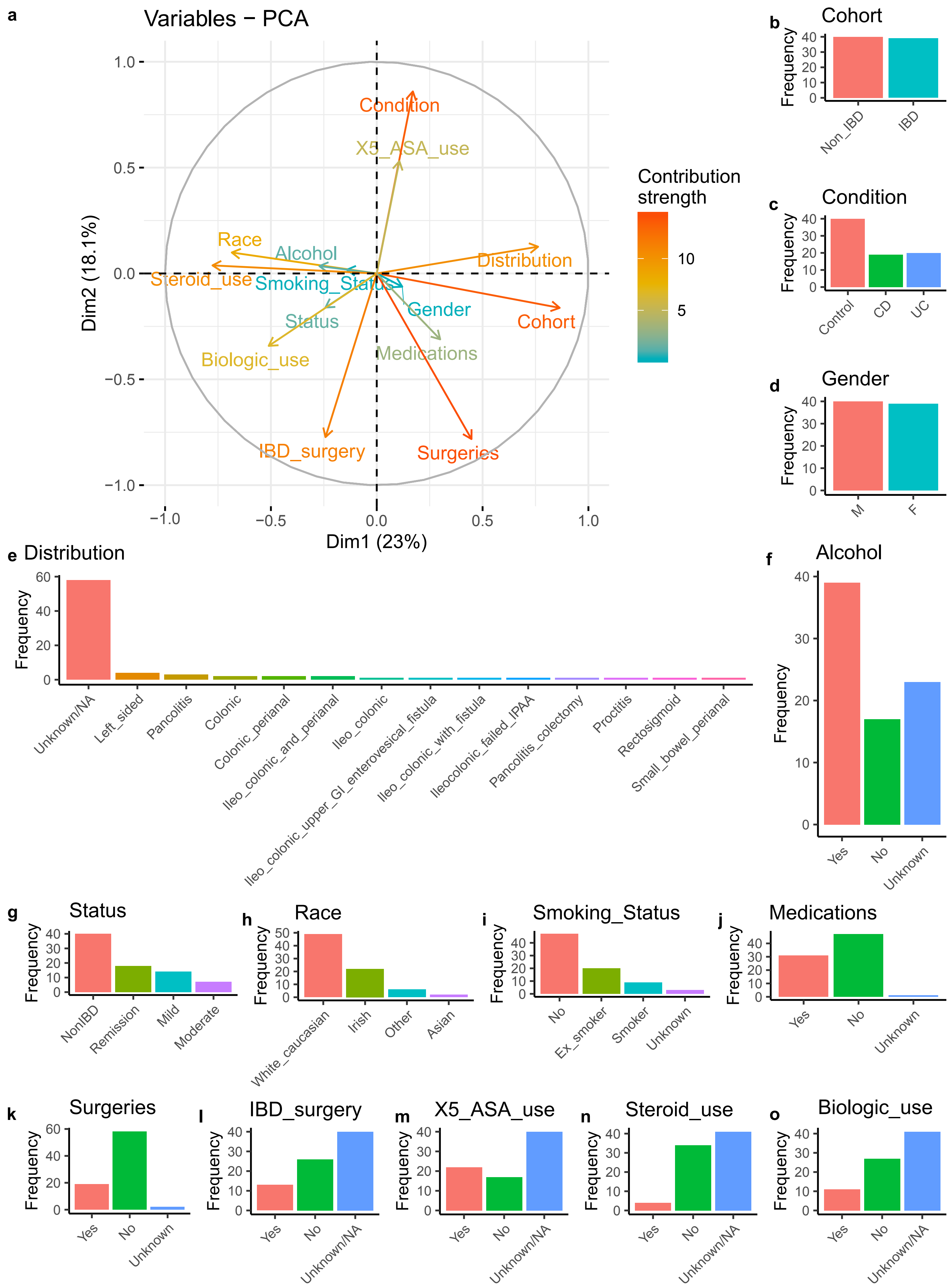

Supplementary Figure 2. Inter-sample variation attributed to specific metadata variables. (a) PCA reduction of 14 variables into two dimensions, with the direction and magnitude of arrows indicating the contribution of variables to inter-sample variation. The frequencies of the 14 variables **(b-o)** associated with the 79 individuals of this study.

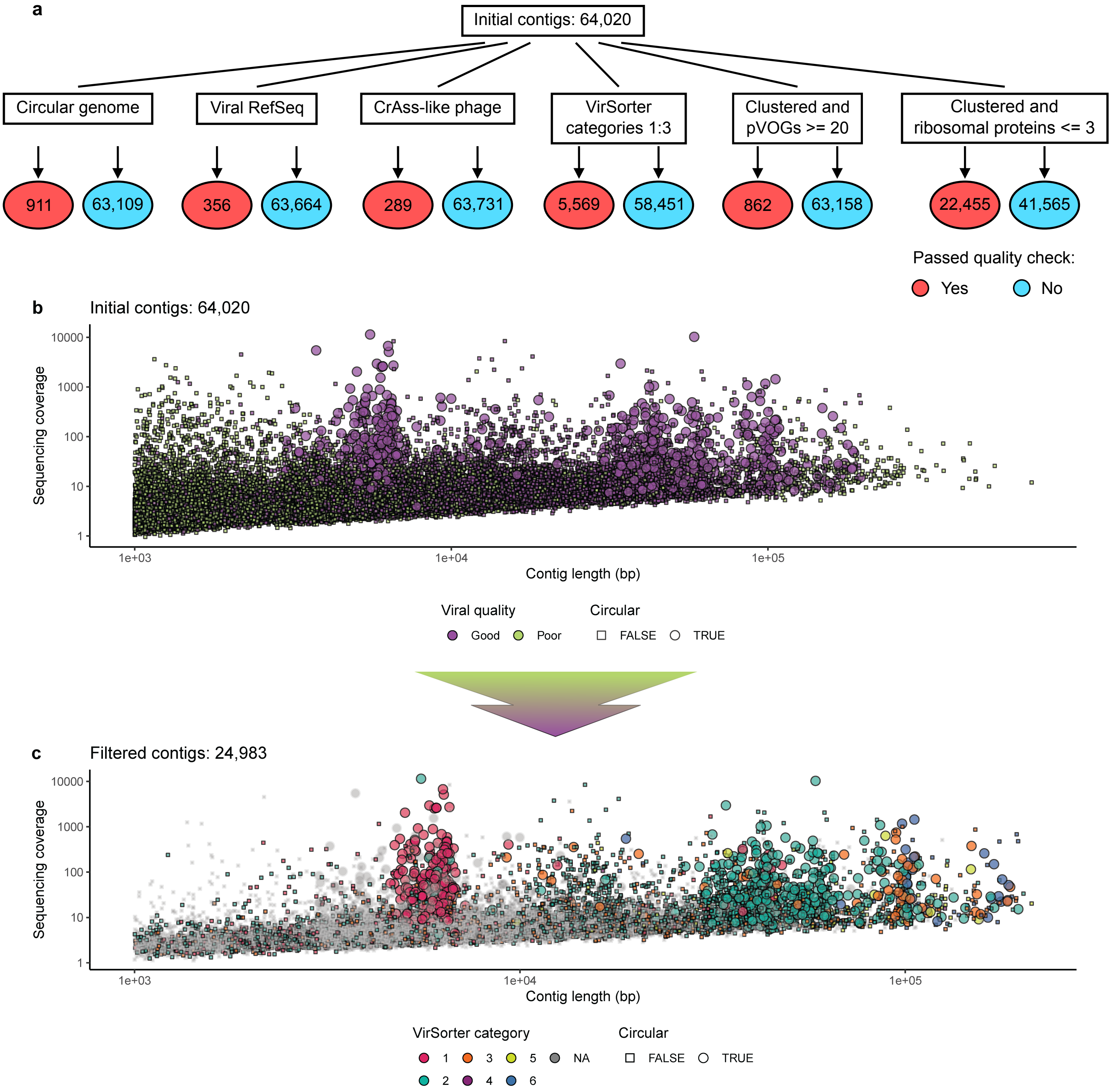

Supplementary Figure 3. Quality filtering of viral contigs. (a) Six filtering criteria were applied to confidently predict human-associated viral sequences. **(b)** The contig length versus sequencing coverage of the 64,020 initial contigs, coloured by their quality-filtering result. The shape and size aesthetic of viral sequences highlight circular genome sequences. **(c)** Contig length versus sequencing coverage of the 24,983 high-quality viral contigs. Each viral contig is coloured by their assigned VirSorter category. Categories 1 to 3 represent the most-to-least confident viral predictions, respectively, while categories 4 to 6 similarly correspond to confidence levels for predicted prophages.

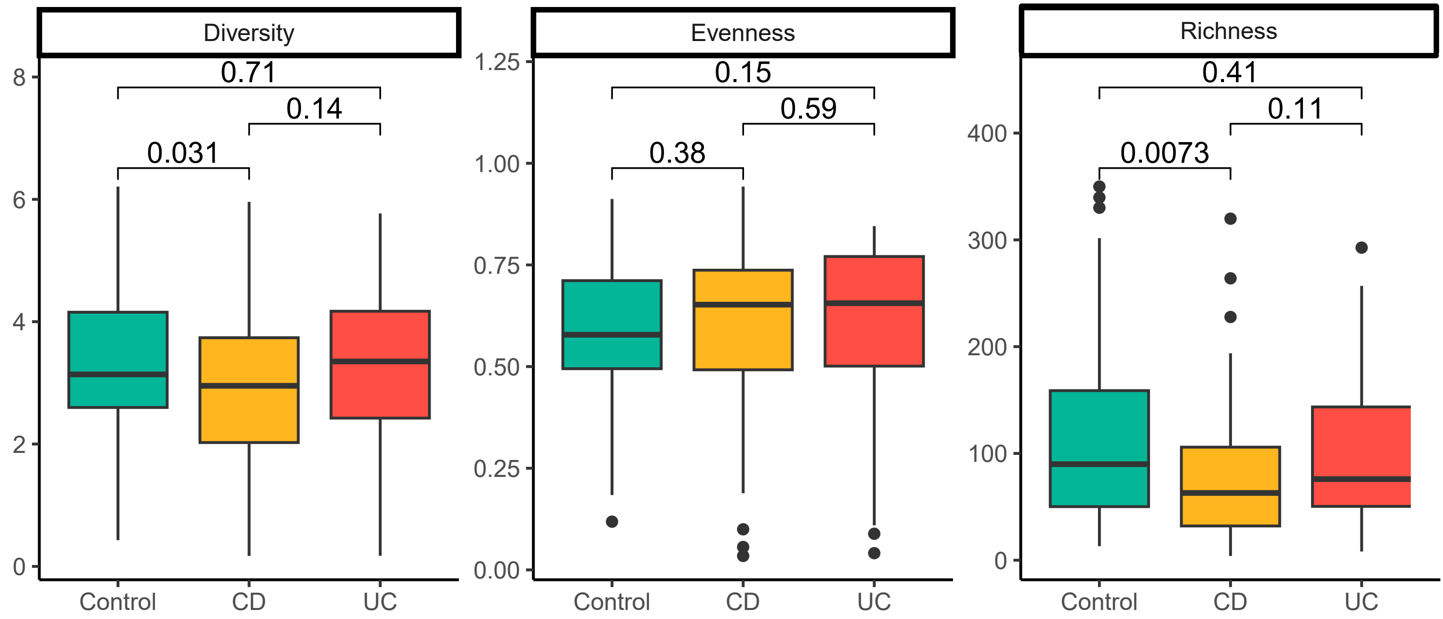

b 16S

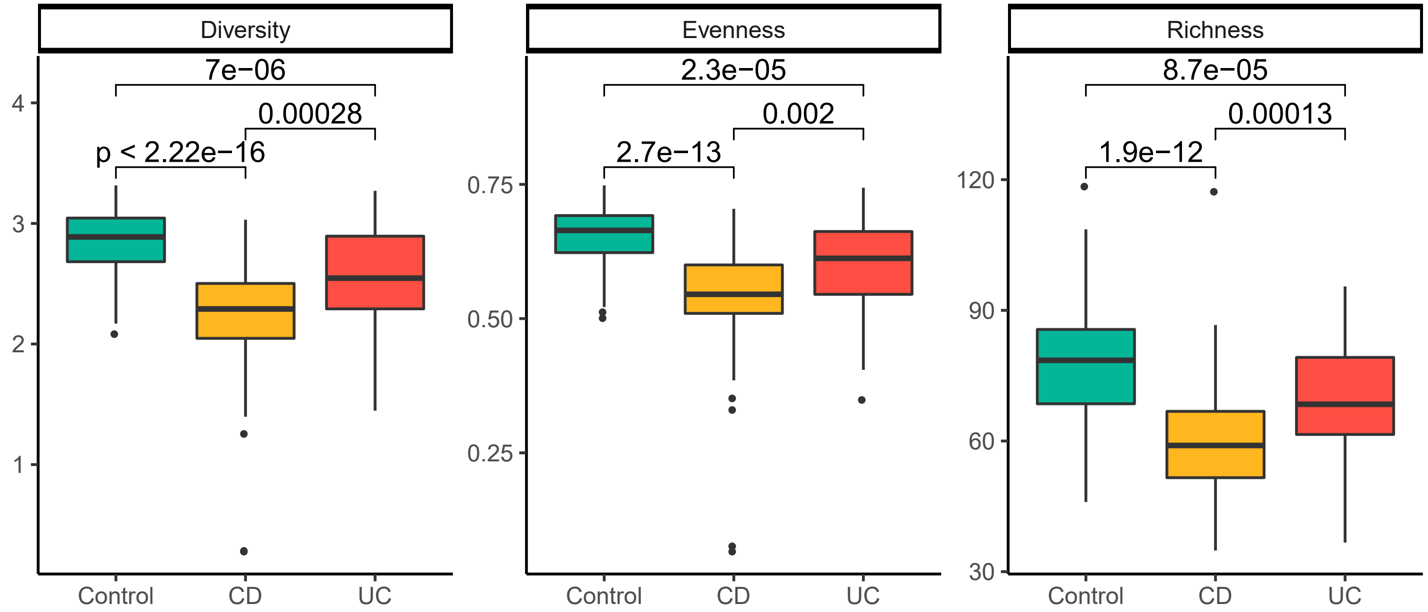

Supplementary Figure 4. Alpha-diversity analysis. The Shannon index alpha-diversity, evenness, and richness values of **(a)** virome and **(b)** 16S faecal sample data. Samples are grouped by IBD condition. Wilcoxon p-values for specific comparisons are shown. Statistics are based on n=118 control samples, n=56 CD samples and n=59 UC samples. Boxplots represent the standard Tukey representation, with boxes representing the 25th, 50th (median) and 75th interquartile range (IQR) percentiles, and the whiskers encompassing values within 1.5 times the IQR.

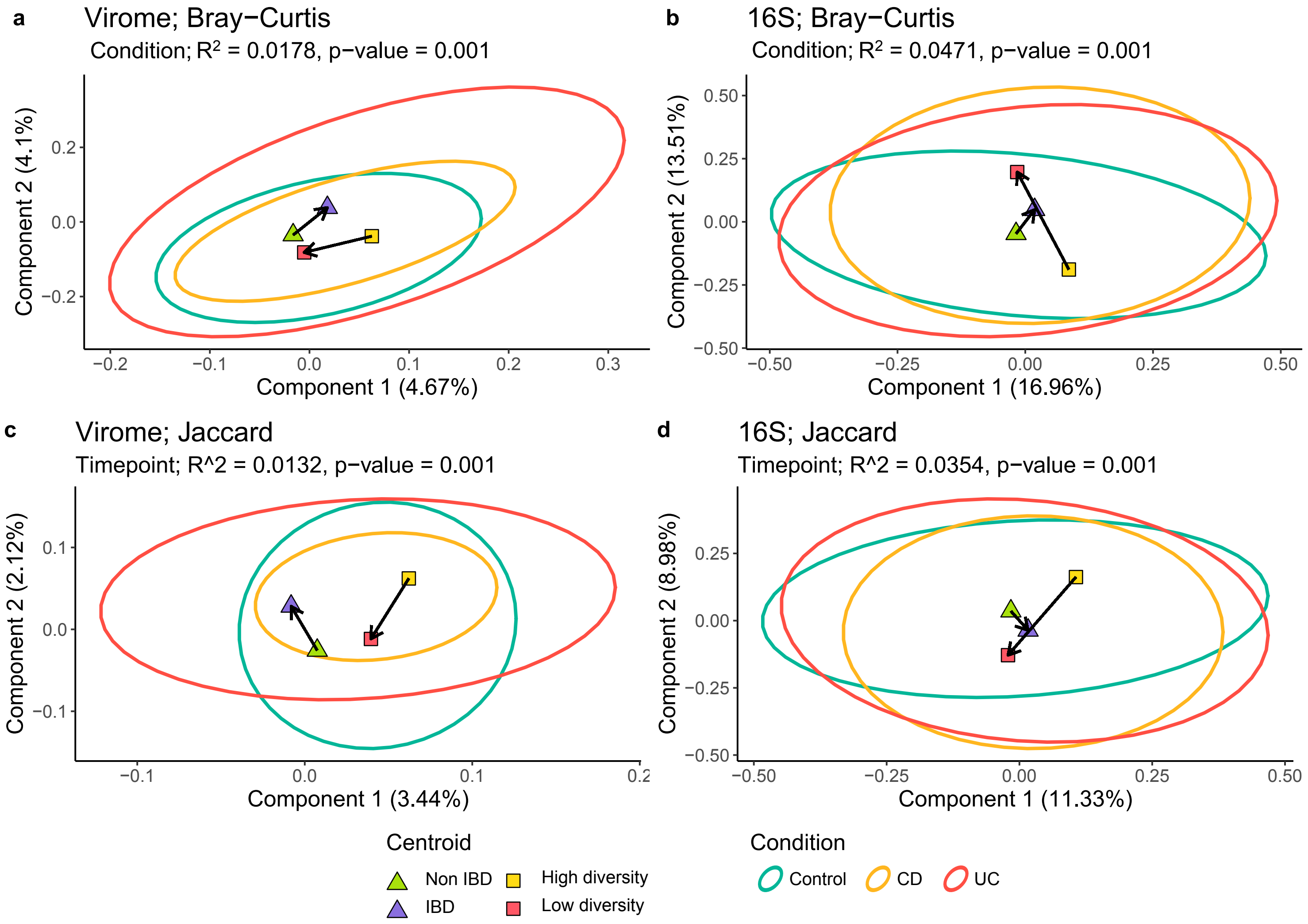

Supplementary Figure 4. PCoA ordination of viral and bacterial beta-diversities. The inter-sample separation of viral and 16S compositions, respectively, using Bray-Curtis dissimilarities **(a & b)** and Jaccard distances **(c & d)**. Each PCoA highlights Control and IBD centroids, and the top 10 most and least alpha-diverse samples.

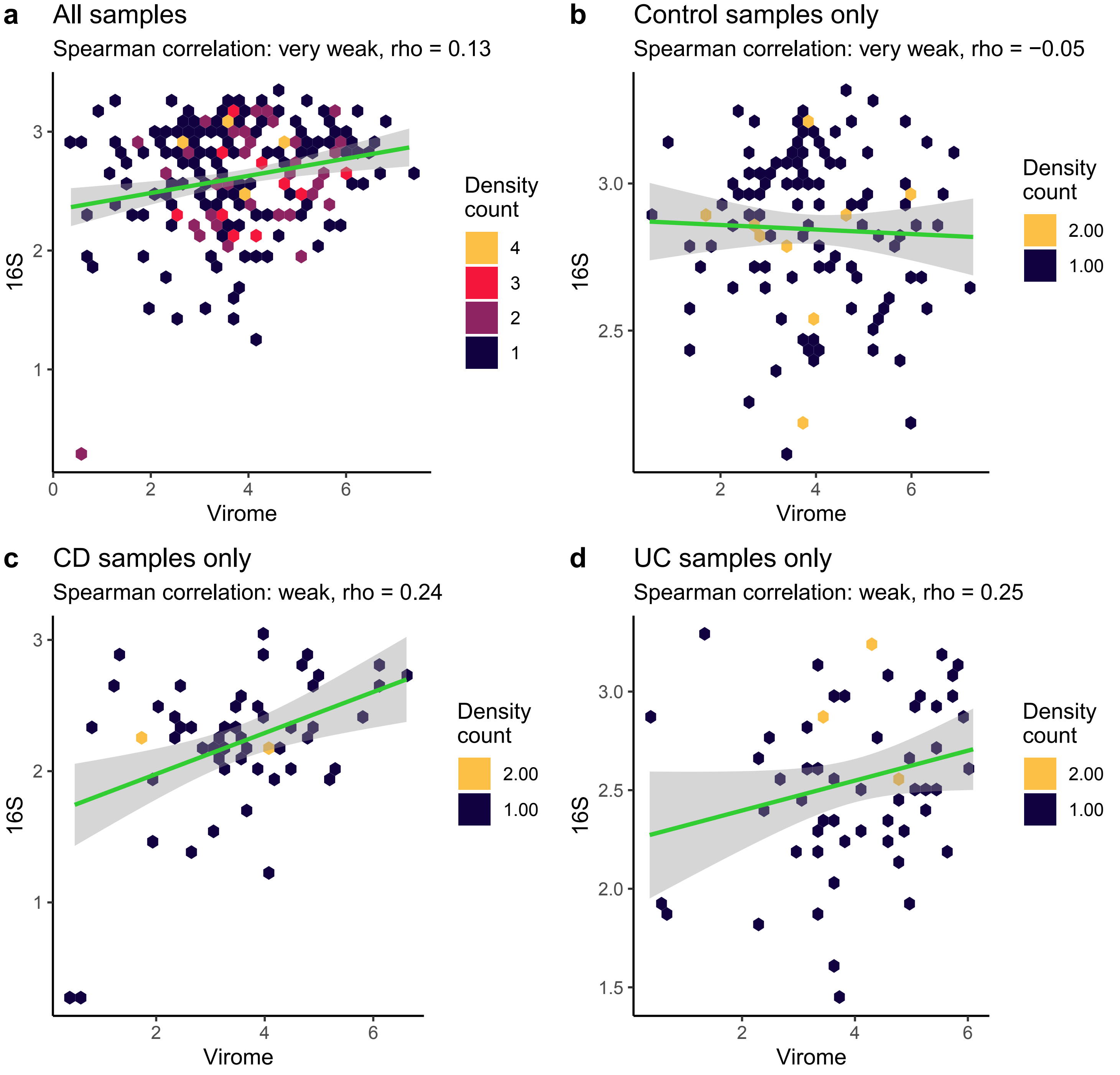

Supplementary Figure 6. Statistical correlation of viral and bacterial 16S alpha-diversities. (a) Spearman's rank-order correlation of all viral and bacterial 16S Shannon index alpha-diversities. Correlations for **(b)** control, **(c)** CD, and **(d)** UC separated samples. Correlation coefficient values, rho, between (+/-) 0.0 and 0.19 were considered 'very weak', while values between (+/-) 0.2 and 0.39 were considered 'weak'.

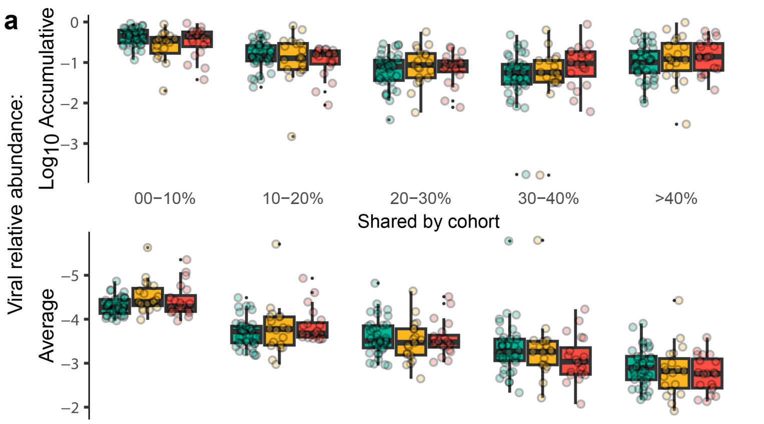

b

Variable	Group 1	Group 2	P−value	P.adj value	P.signif
00−10%	Control	CD	0.008381651	0.13	**

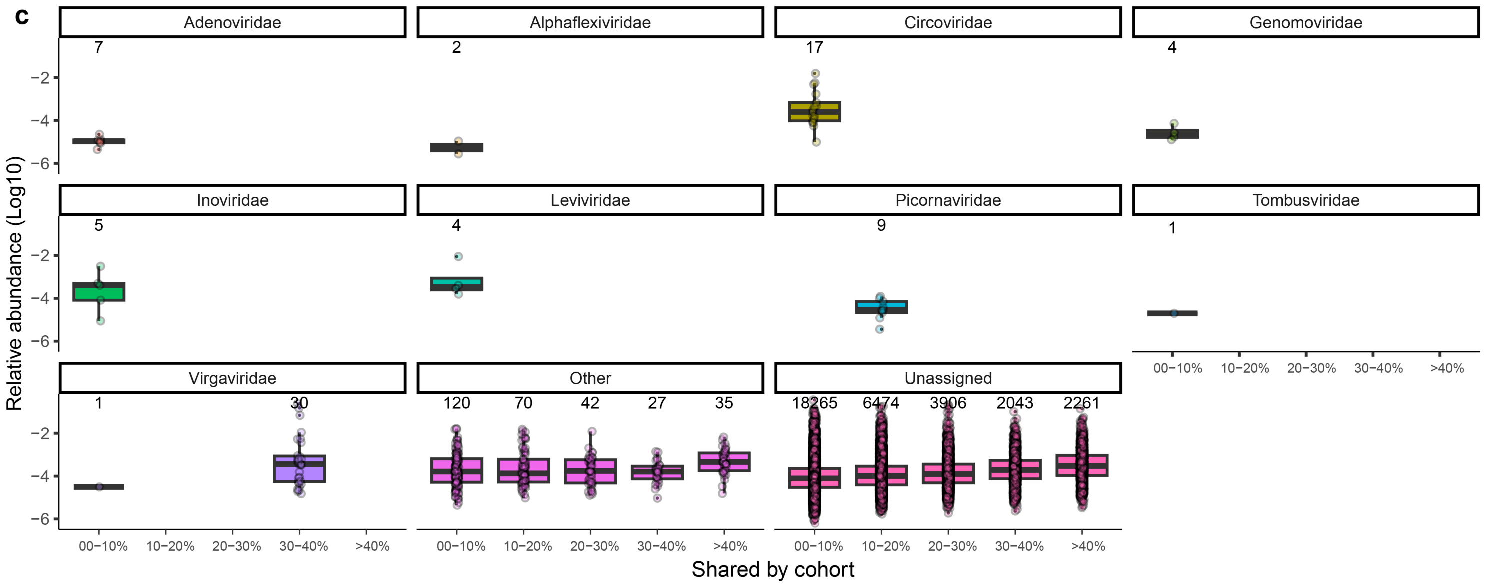

Supplementary Figure 7. Compositional analysis of shared faecal viromes. (a) The relative abundance of viruses and viral clusters shared by varying percentages of the study's cohort (n=79). The accumulative abundance and average abundance of viruses and viral clusters are mirrored, top and bottom, respectively. **(b)** Statistically significant differences in cohort viral relative abundances, grouped by viral sharedness across cohorts were assessed by the Wilcoxon test. **(c)** The relative abundances (log scaled) of infrequently detected, or unknown, viral taxa. The numerical value at the top of each boxplot represents the number of viral sequences. Boxplots represent the standard Tukey representation, with boxes representing the 25th, 50th (median) and 75th interquartile range (IQR) percentiles, and the whiskers encompassing values within 1.5 times the IQR.

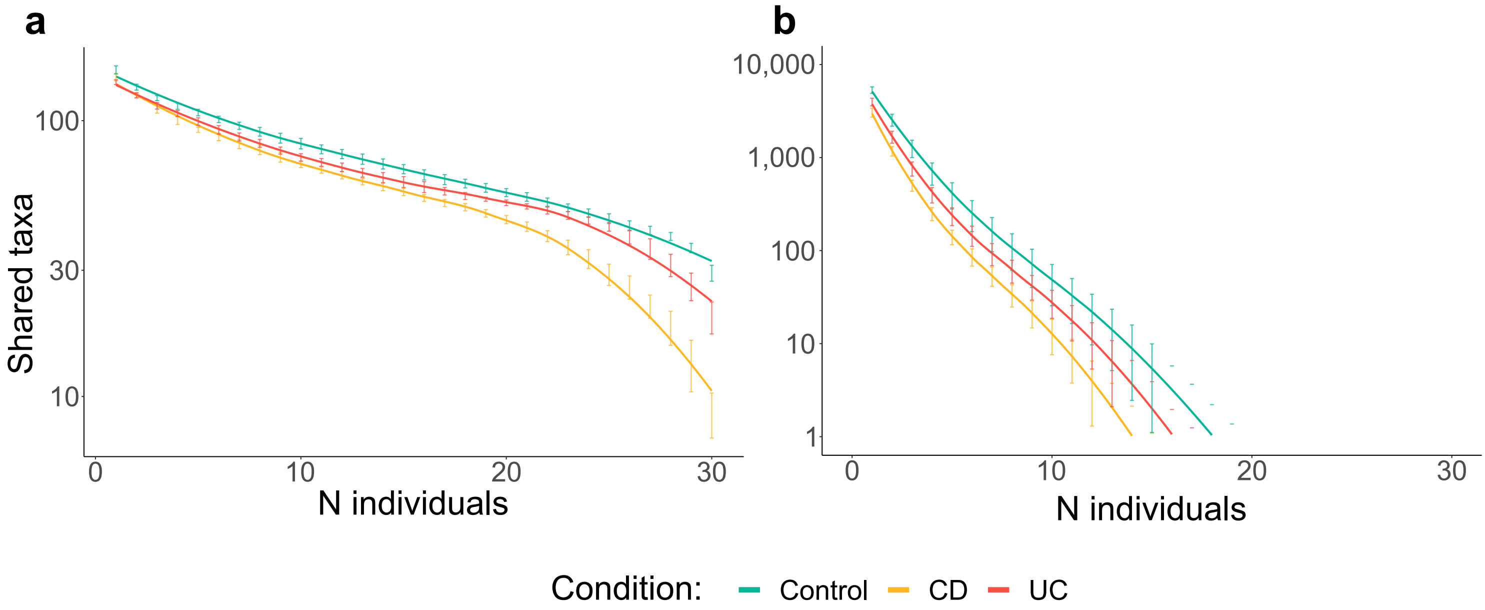

Supplementary Figure 8. Number of (a) 16S and (b) virome taxa shared by an increasing number of the cohort's individuals. Each time point per person was randomly sampled, subsampling was performed 100 times. Each line depicts the mean, with error bars representing +/- the standard deviation.

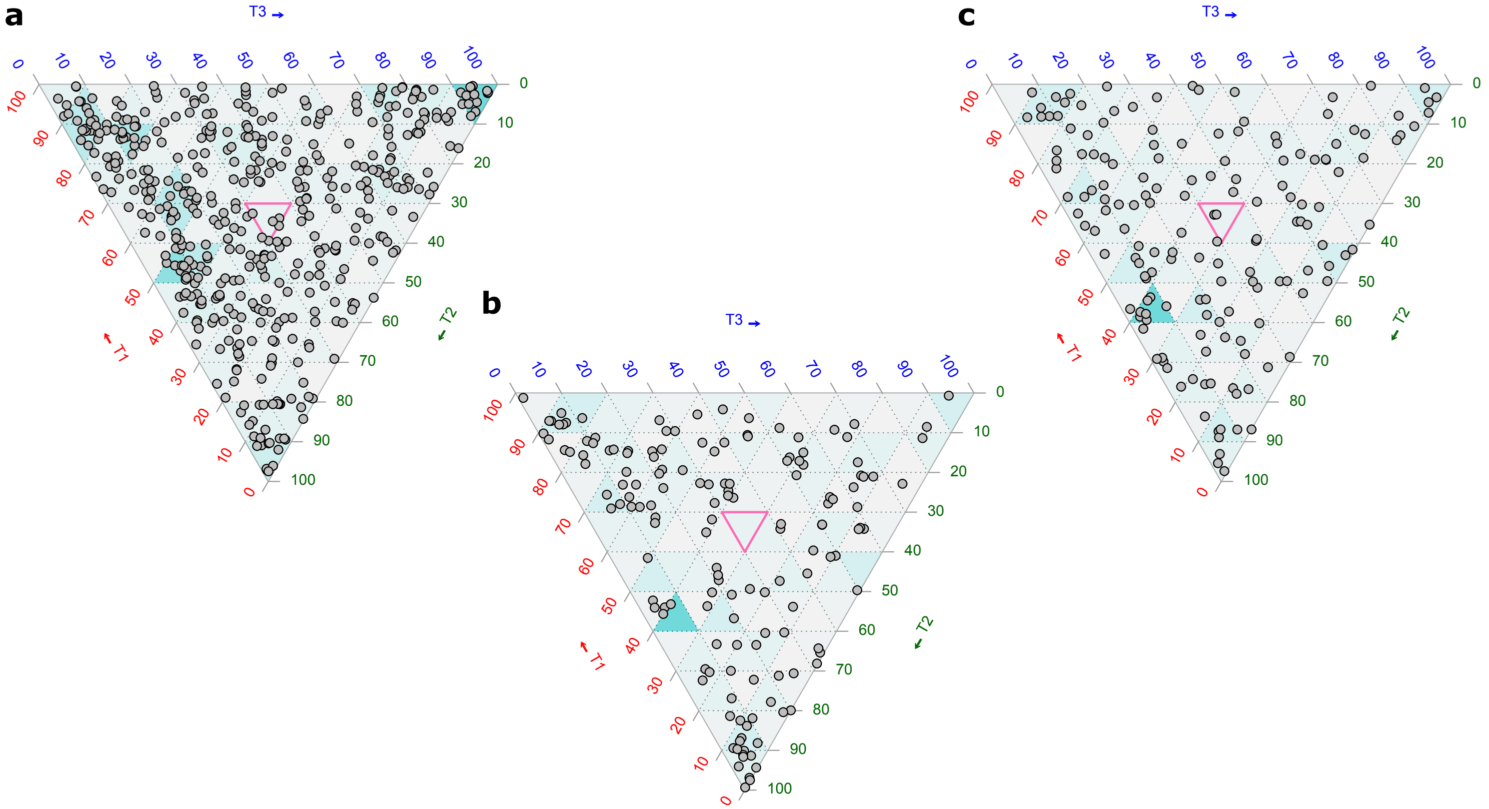

Supplementary Figure 9. Ternary plots of the percentage relative abundance of viruses and viral clusters without taxonomic information, present across all three time points of (a) Controls, (b) CD, and (c) UC virome samples. The central pink triangle of each ternary plot highlights where viruses would be if they were equally present in all three time points analysed.

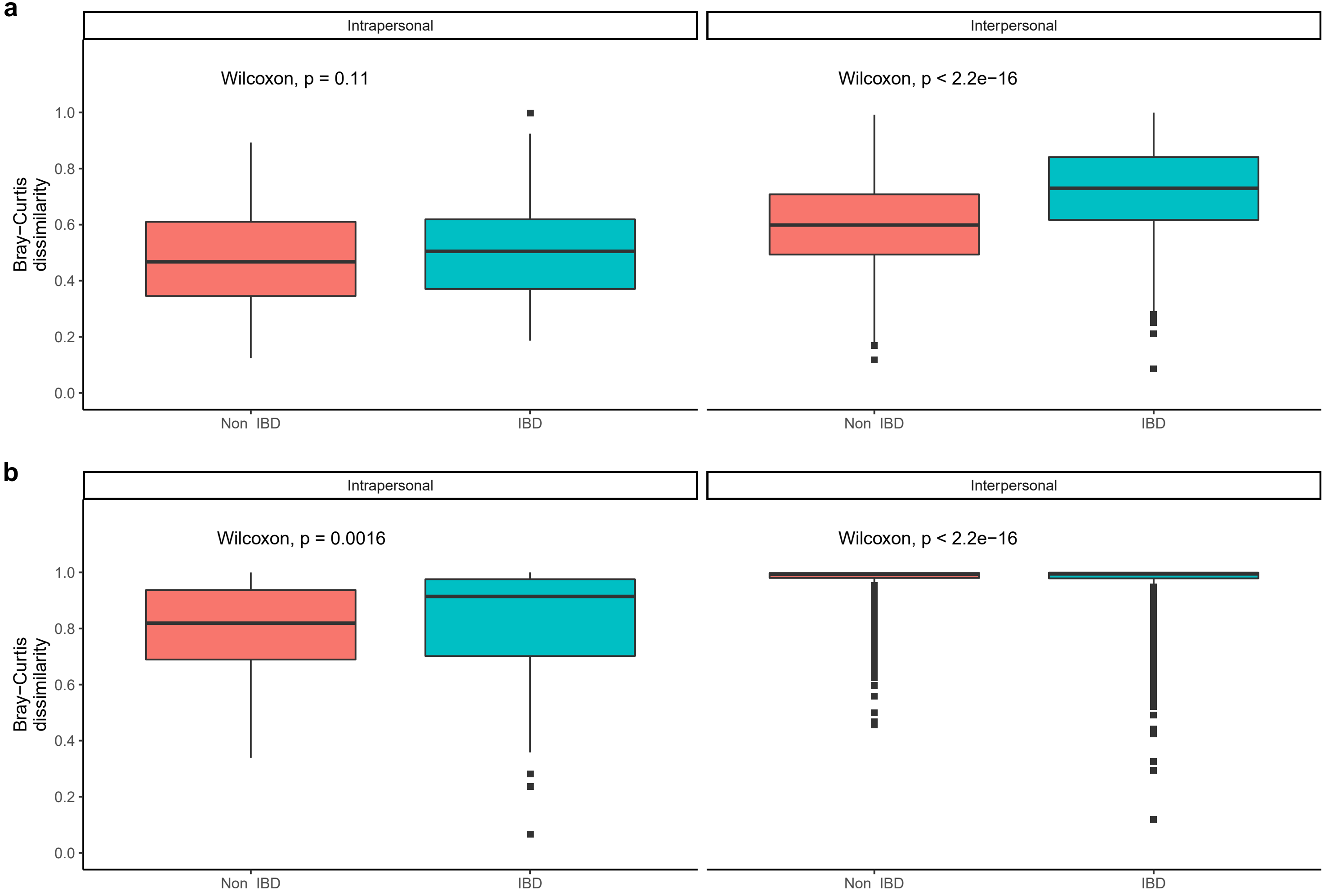

Supplementary Figure 10. The Bray-Curtis dissimilarity between (a) 16S and (b) virome samples, comparing intrapersonal versus interpersonal differences amongst Controls (n=118 samples) and patients with IBD (n=115 samples). Boxplots represent the standard Tukey representation, with boxes representing the 25th, 50th (median) and 75th interquartile range (IQR) percentiles, and the whiskers encompassing values within 1.5 times the IQR.

Supplementary Table 1. Basic cohort descriptive data. Abbreviations: SD, standard deviation; KW, Kruskal-Wallis; CS, Chi-squared; FE, Fisher's exact. All data summaries presented are per person, unless indicated.

Basic details:	Control	CD	UC	Stat. test	p−value	Missing data
Study volunteers<br>Subjects (Samples)	40 (118)	19 (56)	20 (59)			
Age<br>Mean (SD)	36.6 (12.4)	44.9 (11.0)	44.5 (10.6)	KW	0.006	0
Gender<br>Male / Female	17 / 23	12 / 7	11 / 9	CS	0.301	0
Height (m)<br>Mean (SD)	1.7 (0.1)	1.7 (0.1)	1.7 / (0.1)	KW	0.924	17
Weight (kg)<br>Mean (SD)	73.2 (16.6)	80.7 (16)	78 (15.2)	KW	0.225	18
BMI<br>Mean (SD)	24.6 (4.1)	27.4 (3.6)	26.7 (4.1)	KW	0.019	3
Smoker<br>Yes / No	9 / 30	10 / 8	10 / 9	CS	0.021	3
Alcohol<br>Yes / No	26 / 9	5 / 4	6 / 7	FE	0.167	22

Supplementary Table 2. Disease information and activity for patients with IBD. Abbreviations: SD, standard deviation; W, Wilcoxon; CS, Chi-squared; SW, Shapiro-Wilk. Data summaries are calculated per sample, except for "Age of onset", and "Distribution".

Disease activity:	CD	UC	Stat. test	p−value	Missing data
Years since diagnosis Mean (SD)	12.7 (8.9)	11.5 (6.1)	W	0.826	1
Age of onset Mean (SD)	32.9 (12.1)	32.4 (9.8)	W	0.596	1
Remission Samples	37	18	CS	0.01	0
Mild Samples	7	28	CS	3.9E−04	0
Moderate Samples	9	6	CS	0.439	0
Harvey−Bradshaw index Mean (SD)	3.8 (3.9)	NA	SW	9.2E−06	0
Powell−Tuck index Mean (SD)	NA	5.0 (3.5)	SW	0.02	1
Ulcerative Colitis index Mean (SD)	NA	4.06 (2.3)	SW	0.0.27	0
Distribution (n, %)	Colonic (2, 18.2)	Left sided (4, 40)	NA	NA	18
	Colonic perianal (2, 18.2)	Pancolitis (3, 30)			
	Ileocolonic and perianal (2, 18.2)	Pancolitis colectomy (1, 10)			
	Ileocolonic (1, 9.1)	Proctitis (1, 10)			
	Ileocolonic with fistula (1, 9.1)	Rectosigmoid (1, 10)			
	Ileocolonic failed IPAA (1, 9.1)				
	Small bowel perianal (1, 9.1)				

Supplementary Table 3. Medication overview for patients with IBD. Abbreviations: DC, discontinued; FE, Fisher's exact; CS, Chi-squared. Data summaries are calculated per sample, except for "Insulin", "PPI", "Metformin", "Statin", and "Aspirin".

Medication overview:	Control	CD	UC	Stat. test	p−value	Missing data
Steroids Yes / No / DC	NA	8 / 41 / 3	3 / 53 / 3	FE	0.189	4
5−ASA Yes / No	NA	21 / 32	44 / 15	CS	3.8E−04	3
Immunomodulator Yes / No	NA	15 / 38	19 / 39	CS	0.76	4
Biologic Yes / No / DC	NA	23 / 29 / 1	13 / 43 / 2	FE	0.039	4
IBD surgery Yes / No	NA	12 / 5	1 / 19	CS	1.3E−4	6
Insulin Yes / No	0 / 40	1 / 16	1 / 19	FE	0.228	2
PPI Yes / No	2 / 38	5 / 12	3 / 17	FE	0.038	2
Metformin Yes / No	0 / 40	2 / 15	0 / 20	FE	0.047	2
Statin Yes / No	2 / 38	2 / 15	0 / 20	FE	0.249	2
Aspirin Yes / No	0 / 40	2 / 15	0 / 20	FE	0.047	10
Recent antibiotic Yes / No	7 / 109	5 / 48	2 / 57	FE	0.443	5
