# Supplementary material for: Interpersonal variability of the human gut virome confounds disease signal detection in IBD
Source: Commun Biol. 2023 Feb 25;6:221. doi: 10.1038/s42003-023-04592-w (PMC9968284; doi:10.1038/s42003-023-04592-w)
Supplement: Supplementary file 2 — Description of Additional Supplementary Files [file 42003_2023_4592_MOESM2_ESM.pdf]

Description of Additional Supplementary Files

File name: Supplementary Data 1

Description: Description: Contains R Code used for data analysis, and Input files:

1) "Contig_data" = CSV file of contig characteristics;

2) "df_16S_genus" = CSV file of count table of genus level 16S rRNA data;

3) "df_16S_phyloseq" = CSV file of OTU taxonomy from phyloseq with associated sample/patient metadata;

4) "hmmscan_butyrateKinase_prodSelectedContigs_e-5" = Text file of viral contigs containing butyrate kinase domains;

5) "hmmscan_mainCompViral_Ig-like_e-5"= Text file of viral contigs containing Ig-like domains;

6) "metadata_vlp" = CSV file of sample/patient metadata per contig;

7) "otu_legend" = CSV file of OTU abundance and taxonomy;

8) "phyloseq_16S_prop" = RDS file containing OTU taxanomic proportions;

9) "vir_counts" = CSV file of contig abundances per sample;

10) "vir_counts_custom_BOC" = CSV file of contig abundances per sample where the breadth of coverage criteria were met;

11 "vir_coverage" - CSV file of contig

coverage
